# Parental perspectives on consent for participation in large-scale, non-biological data repositories

**DOI:** 10.1186/s40504-016-0034-6

**Published:** 2016-01-20

**Authors:** Kiran Pohar Manhas, Stacey Page, Shawn X. Dodd, Nicole Letourneau, Aleta Ambrose, Xinjie Cui, Suzanne C. Tough

**Affiliations:** Child Development Centre, c/o 2888 Shaganappi Trail NW, Calgary, AB T3B 6A8 Canada; MacKimmie Library Tower, 2500 University Drive Northwest, Calgary, AB T2N 1N4 Canada; Community Member, Calgary, Canada; Child and Youth Data Lab, Alberta Center for Child Family and Community Research, 9925 109 St NW, Edmonton, AB T5J 3M9 Canada

**Keywords:** Consent, Data sharing, Non-biological data, Parents, Pediatric

## Abstract

**Background:**

Data sharing presents several challenges to the informed consent process. Unique challenges emerge when sharing pediatric or pregnancy-related data. Here, parent preferences for sharing non-biological data are examined.

**Methods:**

Groups (*n* = 4 groups, 18 participants) and individual interviews (*n* = 19 participants) were conducted with participants from two provincial, longitudinal pregnancy cohorts (AOB and APrON). Qualitative content analysis was applied to transcripts of semi-structured interviews.

**Results:**

Participants were supportive of a *broad*, *one-time* consent model or a *tiered* consent model. These preferences were grounded in the perceived obligations for reciprocity and accuracy. Parents want reciprocity among participants, repositories and researchers regarding respect and trust. Furthermore, parents’ worry about the interrelationships between the validity of the consent processes and secondary data use.

**Conclusions:**

Though parent participants agree that their research data should be made available for secondary use, they believe their consent is still required. Given their understanding that obtaining and informed consent can be challenging in the case of secondary use, parents agreed that a broad, one-time consent model was acceptable, reducing the logistical burden while maintaining respect for their contribution. This broad model also maintained participant trust in the research and secondary use of their data. The broad, one-time model also reflected parents’ perspectives surrounding child involvement in the consent process. The majority of parents felt decision made during childhood were the parents responsibility and should remain in parental purview until the child reaches the age of majority.

**Electronic supplementary material:**

The online version of this article (doi:10.1186/s40504-016-0034-6) contains supplementary material, which is available to authorized users.

Informed consent is a cornerstone of research involving humans, first codified in an ethical standard in the Nuremberg Code (Rothstein and Shoben 2013). There are three requisite conditions for consent to be considered valid. Participants must be competent to understand the material presented and able to make a decision; they must be fully informed about the purposes, risks and benefits of research participation; and, they must make the choice to participate free from any external influences (Beauchamp and Childress 2001). Though full disclosure of study details by researchers and complete understanding by potential participants may not be attainable, informed consent should not be viewed as a procedural formality that aims to comply with institutional rules. Rather informed consent should be recognized as an effort to respect participant’s decisional authority.

Secondary use of data presents several challenges to the informed consent process (Beskow and Dean 2008; McGuire et al. 2011; Master et al. 2012). When data originate during pregnancy or childhood, complications and concerns arise associated with children’s vulnerability, evolving autonomy, and surrogate decision-making by parents (Ries 2007; Goldenberg et al. 2009; Hens et al. 2011, 2013). Alternatives to the traditional, project-specific consent have been offered, and are elaborated in Table 1 (Master et al. 2012). Consensus is lacking on the parents’ perspectives of preferred consent models, provisions for data withdrawal, pediatric consent/assent considerations, and ongoing communication with repository participants (Beskow and Dean 2008; Master et al. 2012). This lack of consensus produces logistical, financial, ethical and legal challenges for secondary data use. These impediments must be overcome as secondary data use is gaining traction, with most research funders advocating, if not mandating, the sharing and re-use of publicly-funded research data (Canadian Institute of Health Research et al. 2010; Council 2011; Social Sciences and Humanities Research Council 2012). Data sharing processes must be protective and respectful of participants’ preferences to be appropriate and sustainable (Ohno-Machado 2012). Participant perspectives on informed consent for secondary data use would assist policy and practice in this area.

**Table 1:** Consent strategies presented to participants

Consent strategy	Features
Traditional, Project-Specific Consent	Participants are asked for consent for all new uses of their data, including sending it to a repository and every time a new researcher wants to access it.
Broad, One-Time Consent (i.e. Blanket Consent)	Participants are asked for their consent for their data to go to the research data repository just once. If consent granted, all uses are managed by the processes of the repository.
Broad, Periodic Consent	Participants are asked for their consent for the data to go to the repository for future uses but the repository checks back with them at intervals to confirm ongoing consent.
Conditional Consent (i.e. Tiered Consent)	Once in the repository, participants are asked for consent for new uses based on the features of new studies. For example, they may say they only want studies about growth and nutrition, not any other topics. Or, they may only want studies done by students and no other researchers. The repository then uses this guidance to decide how to share that participant’s data.
Opt-Out Consent	Participants are informed that their research data will be placed in the repository and they can do nothing if they are okay with that or they can answer within a given time period to say that they do not want their data to be included.
Notification-Only, No Consent	Participants are notified that their research data has been sent to research data repository and is now available for secondary use. Participants have no opportunity to provide or withhold consent.

To date, the literature has emphasized the sharing of biological data, data originating from a biological sample, which has questionable applicability to the parameters necessary for consenting to secondary use of non-biological data (Brakewood and Poldrack 2013). As non-biological data comprises all information not originating from a biological sample, standards for the protection of biological data may be overly restrictive, wholly inappropriate when used to protect non-biological data. Such concerns would likely impact the costs, logistics and realization of secondary use of data. Previous research with adult participants has generally recognized the need to balance research utility and participant privacy in sharing genetic data (Ludman et al. 2010; Trinidad et al. 2012). Birth cohort parent participants are unique and crucial stakeholders to secondary use as they participate alongside their children in long-term, detailed data collection on myriad constructs (e.g. physical, emotional, developmental and social data) (Golding et al. 2009).

Some studies have directly investigated parents’ perspectives on sharing biological data using focus groups (Halverson and Ross 2012), interviews (Brothers and Clayton 2012), or surveys (Neidich et al. 2008; Burstein et al. 2014; Klima et al. 2014). Parents are generally willing to participate, and allow their children to participate, in biobanks (Neidich et al. 2008; Brothers and Clayton 2012; Halverson and Ross 2012). Parents are generally altruistic in their motives and optimistic in the research implications of biobanks (Neidich et al. 2008; Brothers and Clayton 2012). However, with respect to informed consent, parental views and preferences are unclear. Only one study broached the topic of parental preference on how to be asked for informed consent, but parents were given only one option to discuss: the controversial, opt-out (Brothers and Clayton 2012). Another study found parental surrogate decision-makers were four times more likely to refuse release of their child’s biological data if given the choice, compared to adult participants (Burstein et al. 2014). Subjectively, parents may believe they understand the research context (Burstein et al. 2014); but, more objective studies question whether parents fully comprehend the purposes of biobanks, along with the future uncertainties surrounding data sharing (Klima et al. 2014). For example, parents, like other adult research participants, generally do not understand the indefinite storage of data in biobanks and repositories, the possible risks to participation, and the fact that this research involvement is non-therapeutic (Klima et al. 2014).

Ultimately, more information is needed on parents’ preferences and motivations for consent strategies for secondary use, especially for non-biological data. The purpose of this study is to describe the values and preferences of parent participants from two longitudinal birth cohorts on the topic of informed consent for the secondary use of their, and their child’s, longitudinal, individual-level, non-biological data.

## Methods

Qualitative, descriptive methods were used (Sandelowski 2010). The study population included maternal and paternal participants in two provincial, longitudinal pregnancy cohort research studies: All Our Babies (AOB) (McDonald et al. 2013) and Alberta Pregnancy Outcomes and Nutrition (APrON) (Kaplan et al. 2014). Both cohorts started in 2008 as community pregnancy cohorts that recruited mothers for study during their pregnancies. Combined, AOB and APrON have collected extensive data on child and family health, development, diet and environment from approximately 5200 mother-baby pairs and 1200 fathers (APrON only) at seven time-points. In the current study, participants were purposively sampled from the cohort study participants who had given consent to be contacted for future research (Patton 2002). For maximum variation, key characteristics targeted during recruitment were, mothers and fathers, young (<30 years of age) and older (≥30 years of age) mothers, and both self-reported minorities and new immigrants. This research was approved by the institutional research ethics board.

Groups and individual interviews were undertaken and participants were able to choose their preferred modality (Krueger and Casey 2009). Group interviews were conducted in private rooms at a local tertiary care centre or community centre and individual interviews were conducted via telephone, in private rooms at a local tertiary care centre, or at participants’ homes. Data collection continued until theoretical saturation was reached (Sandelowski 1995). Participants were reimbursed for parking; refreshments were offered at focus groups; and all participants received a $20 gift card to acknowledge their contributions.

A semi-structured interview guide directed conversation during both group and individual interviews. Parental perceptions were sought on general research participation, data sharing and data repositories (7 questions); on governance strategies of data repositories (8 questions); and on consent models for repository participants including consideration of children’s consent/assent (29 questions) (see Additional file 1 for question guide). Along with the question guide, each participant was provided with a Backgrounder document that detailed the types of internal and external regulation data repositories would be subjected to and the alternative consent strategies for participants to consider (see Additional file 2). In this paper, the qualitative analysis will focus solely on consent. The qualitative analysis on parent perspectives on privacy and governance strategies for data repositories are described elsewhere (Manhas et al. 2015).

Interviews were one-on-one between the participant and the lead researcher; focus groups included participants and 2–3 researchers who acted as moderator and co-facilitators (Krueger and Casey 2009). Individual and group interviews were audio-recorded and transcribed. A coding framework was developed iteratively by four research team members using study transcripts and researcher notes (Reed and Payton 1997; Patton 2002). Each researcher read and open coded 6 selected transcripts from which the coding framework of key categories and themes was derived. The framework was then applied by a research assistant to the remaining transcripts. As remaining transcripts were coded, new codes were added or codes collapsed based on consensus of the 4 researchers. Measures were taken to ensure rigour, including fidelity to participant voices and language use; team meetings to reflect on results’ believability; audit trails to track research decisions; and self-criticism by researchers (Milne and Oberle 2005; Patton 2002). In addition to qualitative coding, frequencies of parental consent preferences were recorded.

## Results

Thirty-seven parents participated in this study: 19 in individual and 18 in group interviews (4 groups, 3–6 participants each). Table 2 provides participant demographic information.

**Table 2:** Participant demographics^a^

	Number	Father	Maternal age <30 year	Maternal age ≥30 years	New to Canada in last 5 years	Ethnic minority
Focus group 1	5	1	1	1	1	1
Focus group 2	4	0	Unknown	Unknown	1	4
Focus group 3	6	0	3	2	0	1
Focus group 4	3	0	1	1	0	1
Interview	19	1	7	12	4	3

^a^Some participants fall into more than one category, and hence will be counted more than once in the table. If information was provided on ethnicity, gender or immigration to Canada, then maternal age was not provided by the original cohort, so this information is lacking for some participants

Although this research is qualitative, it is enlightening to view the distribution of responses to the question posed to all participants: “Of the 5 consent models presented, which option would you most prefer?” Table 3 provides the frequencies of these preferences across the 23 transcripts. Most parents prefer the *broad*, *one-time* consent model of being asked once to give blanket consent to secondary use, or the *tiered* model of being asked once at the beginning to consent to secondary use and to give broad parameters on the type of research and researchers that they would want their data to be accessible to. Some parents preferred a combination of the *tiered* and *broad-periodic* models, wherein they would be contacted every two years to be asked permission for continued secondary use and asked, each time, for parameters on the purposes their data can access for. Parents, clearly, do not prefer either of the extreme consent models: *project-specific*, wherein parents are contacted for their permission to share data on a project-by-project basis, or *opt-out*, wherein parents must actively say no to the repository in a 3-month window or else they are presumed to consent to sharing data.

**Table 3:** Distribution of preferences amongst the consent models

Consent model	*N* (%)	Including first choice if more than 1 choice (*N*)	Including first and second choice (*N*)
Project-Specific	1	1	1
Broad, One-time	4	7	12
Broad, Periodic	1	1	3
Tiered	4	7	7
Opt-Out	0	3	4
Mix Tiered + Periodic^a^	4	0	0
More than one Choice^b^	9	n/a	n/a

^a^Participants countered that they preferred a mix of two consent models, the tiered and broad, periodic models, so that participants would be approached every 2 years by the repository and at that time they would be given choices to limit the types of research and researchers who could access their data

^b^Participants offered their first and second choices for the consent model to be used; they did not indicate a single preference. In the case of the focus groups that did not reach consensus (all but 1), this option details the different preferences amongst the group

Two thematic categories emerged from the data: reciprocity and accuracy. Here, we present the combined results of the focus groups and interviews. Results were combined due to the similarities throughout focus groups and interviews.

### Reciprocity: parents want reciprocity among participants, repositories and researchers regarding respect and trust

Parents viewed the consent process through a lens of respect, trust and feasibility. Table 4 contains transcript quotes that demonstrate this theme. First, the five consent models were graded by participants on their perceived respectfulness. Respect was connected to recognition, convenience and control. Parental distaste for the opt-out model related to the lack of recognition of the crucial connection between participants and the data (of themselves and their children). The preferred models for obtaining consent were considered respectful through their inclusion and recognition of parent participants in the decision-making process for secondary data use. *Project-specific* consent was universally lauded for providing the greatest amount of control to participants. However, most participants felt that the high level of contact and involvement required by the *project-specific* consent reduced its convenience, made it overwhelming and annoying, and thus yielded disrespect. The one-time asks of the *broad* and *tiered* models promoted their convenience for participants and repositories, which was perceived to advance their respectfulness.

**Table 4:** Reciprocity-related transcripts’ quotes

Sub-themes	Quote
Respect	
Recognition	“I don’t like [the opt-out consent model] at all. … I feel like …you guys are deciding [sic] for me and I only have three months. Who [gave you] [sic] the right to give me only three months! …There are so many things that people can think when they read this. What if I missed my opportunity? What if I moved and I didn’t get the letter? What if …right? … I wouldn’t like it. …And it doesn’t mean that I wouldn’t participate, but I would think twice…” [Mother, interview 6]
	“Well [sic] it might feel good to have that power, right. To be asked. We always like to be asked and then say yay or nay. So, ultimately I don’t think [the project-specific consent model is] a great idea, but I think it would make us feel good”. [Mother, focus group 2]
Control	“Well [the project-specific consent model is] definitely good for people who are concerned. … [sic] To know that you’ll be directly contacted if anyone wants to use your information, would give people peace of mind. … I think for confidentiality like it would probably put her mind at ease knowing that she’ll be contacted personally”. [Mother, interview 3]
	“It’s more secure because every time you can opt in or out depending”. [Mother, focus group 3]
Convenience	“Sometimes having too many options… I’m just going to say [it]. We have so much paper that comes through: daycare and [sic] preschools. And it’s just all coming through and you’re like “ahh yeah what’s this about even again?” So … it’s keeping it simple. … You want to give people options because you want to make sure we’re respecting everybody’s beliefs and everything. But… I don’t even know what really [the] possibilities [are for] the pharmaceuticals?… I’m still a big fan of the, what is it the second choice, the broad one”. [Mother, focus group 4]
	“[Asking for] consent every time … might be, depending on the number of researchers and that’s something I wouldn’t really know anything about, [but] that could be sort of overwhelming and kind of irritating … constantly having requests like “can I use your data today”. And then you get [sic] … four researchers asking next week. … And that might be irritating. [Mother, interview 5]”
Trust	
Consent as information-sharing	“I would like, if I trust you [sic], I would like to know, if you asked me. It can be one time. But [sic] if you decided it’s good someone for future [use] someone decided it’s good, like someone good, yeah you can [share]”. [Mother, interview 18]
	“They would just be annoying. … I would want to be contacted now to say yes, we’re setting up this data bank and would you like your data included and that sort of thing and this is how it would be used and there would be a committee that would review this and it would go through ethics boards and I would like that explanation. … But, I definitely wouldn’t want to be contacted [for every project], I just think [sic] it’s overkill”. [Mother, interview 17]
	“[The broad, periodic consent model] would be interesting, … every two years, if they’re asking for consent and let [the parent] know how your data has been used or how many studies it has been used. … [I]f it was me, [sic] it would make me feel good [sic] that [sic] something that I’ve done is [sic] actually helping people”. [Mother, Interview 1]
Recognizing parent decisions	“Yeah [sic] I think [sic]… if you contacted [the child participants] when they became an adult or whatever, that would be another logistical [issue]. I think, as a parent, you just trust that. You just trust the consent of the parent when the child was a child”. [Mother, Interview 3]
	“No, I don’t think [the child should be re-contacted or re-consented], not at all. … Because, they didn’t make the decision in the first place, [sic] their parents [did]. [sic] The participant was a mom who gave out the information, so [sic] no, I don’t think [the child] should be [sic] contacted, no. It’s data. … It’s for the good of everyone. So, I don’t think they should be contact[ed]”. [Mother, Interview 2]
	“You would never think that a parent or a guardian would do something in their best interests, that isn’t in the best interests of their child. And I don’t see how giving their data like [this] would ever benefit the adult. [sic] I don’t think that would ever be taking advantage of the child”. [Mother, Focus Group 1]
Feasibility	
Logistics	Sometimes the [range] is really wide and then if you’re going to say this is [sic] strictly health or strictly [sic] education, stuff like that. [sic] But it can be still used for other things. … Using it as a correlation between certain things in the future that we don’t even know or don’t think about right now so. … It will be [sic], just to store it in the database a certain way… a lot of work and money”. [Mother, Interview 16]
	“Unless it’s sort of one of these sheets that you can kind of feed into a machine to let it know who is consenting to what type of research [using the *tiered* model] that might be again logistically difficult to implement”. [Mother, Interview 5]
	“You guy’s side [sic], whoever or any of the researchers, … [*broad* consent is] less cost than them going and getting new data themselves, but it is still a cost that seems unnecessary to try to follow up and contact each individual person for their data. [sic] It would be a lot of time and cost on their side and then for the person being contacted. [sic] I don’t know how many times [I would be contacted]. … Cause if I was being contacted weekly or even monthly, I think I would get irritated”. [Mother, Interview 11]
	“[*Broad* consent is] definitely more efficient for you guys. You don’t have to keep contacting the people, less administrative stuff”. [Father, Interview 14]
Point-of-contact	“I’m just wondering maybe if the secondary users want to contact somebody that it actually doesn’t come from them, but comes from the original source. If that makes sense … one point of contact, instead of multiple”. [Mother, Focus Group 3]
	“So, I wouldn’t agree that if they shared my information, my address and name. [sic] What’s connected directly to you guys, like, to [the original cohort]. I’d really like you guys [to] contact me to update something but just you guys”. [Mother, Interview 2]
	“I think it could be a possibility certainly. … I don’t know if I would want to be contacted directly by the secondary researcher. I think I would want to be contacted either by [sic] the data bank itself or the repository or whatever we’re calling it. … But I do think that let’s say a secondary researcher is using the data and they realize you know it would be really helpful you know to look at these kids now or to talk more to the parents or something. I wouldn’t mind having an ability to opt in [sic] to that kind of study but also to opt out and to not have to [sic] feel pressured. … I mean obviously researchers are not supposed to pressure people, but I mean I think I would feel less pressured if I was contacted by a neutral body rather than the researcher themselves”. [Mother, Interview 17]

Second, by asking permission to share data, trust could be built; and, by recognizing the permission provided, trust could be sustained. The informative aspects of the consent process, no matter the model, were valued by all parents as a way to know about (a) the research potential of their and their child’s data; (b) the security and governance processes around the repositories that would facilitate secondary use; and, (c) the contribution the data actually made to the greater good. By asking for consent from parent participants, most parents felt increased trust in the data repository and the data-sharing enterprise. Many parents viewed trust as moving in the other direction as well, with an expectation that repositories should trust the parents in their decision-making. This sentiment arose particularly when parents were asked if and when their children should be involved in decision-making for long-term storage and sharing of the data collected during childhood. Four parents and one focus group indicated that parental decisions made during childhood should not be revisited; six parents and three focus groups suggested that children should not be involved in decision-making until they were closer to the age of majority (i.e. 18 years). Four parents offered ages in the 11 to 14 years range as when children should begin to be involved and heard with respect to their preferences on the re-use of their data. Two parents suggested involving children from an early age (e.g. 2 or 3 years) in the research participation and decision-making. Most parents view decision-making about their children as strictly their purview, especially for the 18 years when the children are legally in their stead.

Finally, parents recognized that the projected size and longevity of secondary use would impose feasibility implications on garnering and maintaining consent. Logistically, the time, costs and realizability of re-visiting consent (i.e. *project-specific* and *periodic* models; returning to children as they grow for consent; or further, new research) were seen to be significant hurdles. Parents also recognized that the implementation of parent preferences via *tiered* or *project-specific* consent would also introduce bureaucracy, costs and limitations that would slow or hinder access to data. Many parents recognized these hurdles as an unnecessary hardship for the repositories in the pursuit of balancing respect for participants against the goal of advancing society. Parents also questioned the ability to maintain contact information for families in the long-term that recognized mobility as well as confidentiality. Parents recognized that re-visiting consent could conflict with parental desire to have only one point of contact between participants and the secondary use enterprise: the original researcher.

### Accuracy: parents worry about the interrelationships between validity of the consent processes and secondary data use

In the context of informed consent, accuracy emerged as a concern of, and about, parents. These concerns centred on the process of consent and the implications of providing or withholding permission to re-use the data. Table 5 contains the transcript quotes that evidence this theme. First, parents worried about the inaccuracy implicated by expected loss to follow-up with the *broad-periodic*, *opt-out* and *project-specific* consent. Family mobility, or family annoyance at frequent consenting, could lead to repositories being unable to ask the consent question, or could lead to parents withholding their consent for secondary use, respectively. Existent data would become unavailable for secondary use, and could not contribute to the greater good. This was a major disadvantage for the *opt-out* model as parents worried that parents may not receive a notification in time, and their silence would be misinterpreted as a passive consent when in actuality it was the anti-thesis of informed consent: uninformed and involuntary.

**Table 5:** Accuracy-related transcripts’ quotes

Sub-Themes	Quotes
Consent process concerns	
Loss to follow-up	“But, the disadvantage [of project-specific consent], and I think it’s too big of a disadvantage, is that you could lose people to follow-up because you may not be able to contact them every time [that] the secondary researcher wants to use the data. And it’s time consuming. And it’s expensive to have to contact these people every time you want to use their data. So, to me I don’t think that this is a viable option for the data library and I’m sure it’s not probably the option that will be used”. [Mother, interview 19]
	“Cause they could [sic] could move or it could be mailed to the wrong address or [sic], I don’t know, lots of things could happen. I think it’s better to have … their consent rather than them not saying anything is their consent”. [Mother, Interview 3]
	“Because I think you can lose contact information from people. … You get a new cell phone. [sic]… You move to Toronto. [sic] I think eventually within 10 years, you probably are not going to be living in the same place or whatever”. [Mother, Focus Group 1]
Lack of understanding	“In the sense that you guys are saying you don’t know where [the data is] going to, so to consent [broadly] … [sic], unless you know where [the data is] going to, [the consent question is] kind of a moot point I think”. [mom, Interview 13]
	“Well disadvantages is that [the parent] would have to have way more knowledge about who [can] access that information of not. My goodness, could you imagine the time for that? … You would need to know more. I mean [sic], if there was a committee who could determine who could use [the data] that would be better, because they have more knowledge why this person needs the information. But from our position, from my position I’m thinking well I’d be saying yes [to share] but really without knowledge I really don’t know what I’d be saying yes to really”. [Mother, Interview 15]
	“I think [sic] the disadvantage to [the *tiered* consent model] is … for any researchers thinking outside the box. If what they’re doing is not covered in the preferences, then the data that could be used is then not available”. [Mother, Interview 7]
	“A negative part would be to make sure that everyone understands [sic] how that data is going to be managed and what the process will be to access that data, and who can access that data. If that’s clearly outlined and then if the understanding is there from all of the participants, [both] would [sic] maybe [be] more difficult or to assess”. [Mother, Interview 10]
Concerns about parents	
Consent vs. Withdrawal	“If a parent had forgotten, but they really would like to opt out, [sic] then, it reminds [the parent] [sic] that they are opted in and can change their mind. But … I kind of think … it’s the parent’s responsibility [sic], if they agree to stuff like this [sic], to keep it in mind. … If they just change their minds, it’s on them … to [sic] take that step”. [Mother, Interview 7]
	“I think if every two years it came up and say “Hey all of your data was used for this research in the past two years, here’s some potential stuff that could be coming”, that gives you an idea of what else could be researched. [sic] You can continue to consent to this. I think that would have a lot more information on it and it would make a little more sense to do that one. … You’re going to contact me and I’m going to say “Hey you, your research was used in this, and this and this and this and you know what do you think?”… “Do you want to keep going or not?”” [Mother, Interview 13]
	“The good thing is that [sic] the data is there for two years, and then if [sic] something happens or the person changes their mind, every two years is a good amount of time to get whoever is doing the research to use that data. … It’s a reasonable amount of time and if you want to change your mind, you’re not locked for life. [sic] You can [sic] say for the next time, no thank you very much [sic] I don’t wish to participate and then your information can be taken down”. [Mother, Interview 16]

Second, parents questioned the sufficiency of understanding that parent participants would have to make long-term decisions about the re-use of their own, and their child’s, research data. For some parents, this concern manifested as a result of the future uncertainty of secondary use for all consent models except the *project-specific* consent model. For other parents, this concern connected to a lack of understanding of the research realm. Participants questioned the ability of parents to understand the full implications of exercising choice in the *project-specific* or *tiered* consent models. Parents expressed overwhelming support for their data to be shared and to contribute to the greater good. Parents worried that, if they gave limitations on what type of information could be shared or on what type of researchers could access their data (e.g. academic versus industry researchers), they would inappropriately interfere with making the data as widely accessible and useful as possible. Parents worried that valuable data would be lost, or that important research would not be completed due to inadvertent or uninformed prejudices against the research type or environment.

Third, there seemed to be confusion and conflation around informed consent and the right to withdraw amongst parents. For some parents, it was simple confusion that required researcher clarification that no matter which consent model was chosen, participants were free to contact the repository at any time to withdraw. However, other parents viewed the consent processes and models as modes to keep up-to-date and to be prompted to reconsider their original consent. Parents were evenly split in allocating the responsibility for keeping up-to-date and to reconsider consent. Those parents who created the mixed model of *tiered* and *broad* consent generally wanted more control in their consent, but wanted the responsibility of maintaining the currency of the consent to fall to the repository. Meanwhile, those parents who preferred the *tiered* or *periodic* consent models, felt parents were responsible for staying current on how their, and their child’s, research data was being re-used and for re-assessing their status on their permission.

## Comments

Parent preferences for the informed consent process to re-use their, and their child’s, non-biological data mirrors that from the literature on sharing biological and genetic data. Parent participants want their data made available for secondary use and they trust researchers and institutions; however, parents still want to be asked for permission for re-use of their data (Beskow and Dean 2008; Ludman et al. 2010). The findings here also support previous findings that participants usually understand the tensions amongst the various consent models, especially as they relate to costs, feasibility, and accuracy of the consent and of secondary use (Beskow and Dean 2008; Ludman et al. 2010; Trinidad et al. 2012). As indicated in previous research, parents generally understand research, but appreciate that there are limits to their understanding about the nuances of research aims, methods and implications (Klima et al. 2014).

Novel findings centre on consent preferences, especially among parents. The exact preference amongst consent models has thus far been unclear as it was highly dependent on the options offered to respondents (Beskow and Dean 2008; Ludman et al. 2010; Trinidad et al. 2010, 2012). The *tiered*-consent model was found popular, although other studies found support for opt-out and broad (one-time and periodic) consent models when these were discussed alone or in conjunction with tiered models (Beskow and Dean 2008; Ludman et al. 2010; Brothers and Clayton 2012; Trinidad et al. 2012). Our qualitative research illustrates the rationale behind consent preferences, and narrows the field given the breadth of consent options discussed with parents. Parents do not want extreme consent models when asked for consent to share already collected research data. *Project-specific* consent is too taxing, for parents and repositories; *opt-out* consent risks inaccuracy and does not feel respectful or involving enough for parents. These findings counter previous research, which asked parents about only one consent model, *opt-out* (Brothers and Clayton 2012). Parent preferences amongst the remaining models were relatively evenly split. However, parental concerns for the accuracy and logistical challenges of the *tiered* and *periodic* consent models suggest that the *broad* consent model would be most preferable for parents. Though *broad* consent for the secondary use of previously-collected data can still be a large endeavour, parents feel it would be a minimum requirement for maintaining trust in research and secondary use. Some concerns have been raised that informational research becomes biased if consent is relied upon as those who would consent to sharing data could be different from those who do not consent (Rothstein and Shoben 2013). Statistical research confirms, however, that the claims of the amount of consent bias are likely overstated; and any residual effects of consent bias fall below acceptable levels of imprecision (Rothstein and Shoben 2013). This research could be extrapolated to suggest that the use of *broad* consent would be acceptable for current and future research projects, which plan for secondary use before data collection begins. In those cases, the logistical issues would be negligible.

Another novel finding relates to the discussion on how to involve children in the long-term secondary use of data collected prior to birth and during their childhood. Most parents felt that the decisions made during childhood fell to parents to make, and should remain in parental purview until the child reached the age of majority. A minority of parents wanted to involve their child along the research process, influencing the interpretation of the extant literature. Researchers suggest that age is not a good marker for child capacity in research decision-making (Gibson et al. 2011). Parents seem to concur and feel that secondary use can be solely decided by parents. Parents associate their decision-making to the best interests of their child. This parallels the recent findings that parents are much more reticent to share their child’s biological data if given the choice, compared to adult participants (Burstein et al. 2014).

Two limitations challenge this research. First, this research involved a complex, uncommon topic for participants. The novelty and complexity of secondary use and consent models required detailed background information be provided to participants during data collection. The interviewers and focus group moderators struggled to appropriately explain this topic without overly influencing participants. During data analysis, sections of a minority of interview transcripts were considered unusable as participants’ responses were affirmations or reiterations of the researcher explanations. Focus group transcripts did not contain such disutility. This challenge does not invalidate these findings because (1) no interview transcripts were complete reiterations; (2) interview and focus group transcripts were comparable in thematic findings; and (3) theoretical saturation was reached well before the completion of data collection. The second limitation is that focus group transcripts could not be transcribed to preserve participants’ unique voices. Four transcriptionists attempted to transcribe, but none could consistently isolate participant contributions for the focus group duration. Thus, the interactional components of the focus group discussions could not be analyzed (Morgan 2010). The focus groups remain fruitful discussions that did not reiterate or affirm of focus group moderator’s comments. Field notes and observations revealed that the discussion and interaction in focus groups made more participants change their minds, compared to during unidirectional interviews. The topic’s complexity and uncommonness was alleviated in focus groups, compared to interviews. The 19 interview transcripts and 4 focus group transcripts were analyzed together, which aligned with the use of the same semi-structured question guide.

The planners and implementers of secondary use of non-biological data from families must consider parent perspectives, or at least evidence about parents’ perspectives, in their approach to the issue of consent. Parent research participants wish to share their, and their child’s, non-biological research data. However, they would like to be informed about the potential for secondary use, and want to be asked for permission before such sharing occurs. Consent demonstrates respect and builds trust, but is challenged by attrition and sufficiency of parental understanding of long-term consent.

This research project began as a stakeholder engagement project to compliment the organizational efforts to establish a child-focused research data repository in Alberta, Canada: the Child Data Centre of Alberta (Alberta Centre for Child 2013). These findings will inform how this repository will direct and support primary researchers who plan to deposit research data into the repository. These findings informed the design of a web-based survey of a broader proportion of these two birth cohort populations, which will offer more generalizable findings on consent model preferences. Further research is required, however, on other stakeholders’ perspectives, such as researchers and adolescents, on sharing non-biological, pediatric data. Understanding adolescents’ perspectives would offer an important contrast to the parental views to not involve children as they age in secondary-use decision-making. By understanding how researchers share their primary research data, logistical realities and current practice can be compared to participant perspectives. By understanding the distance between how parents want to be involved and asked for permission compared to how they are actually involved and asked for permission, steps can be taken to promote the reciprocity and accuracy of the consent process for secondary use.

## Additional files

Additional file 1:
**Question guide.** (PDF 323 kb) Additional file 2:
**Backgrounder document that detailed the types of internal and external regulation data repositories.** (PDF 210 kb)

## Abbreviations

AOB: all our babies
APrON: Alberta pregnancy and outcomes nutrition
