# Supplementary material for: Parental perspectives on consent for participation in large-scale, non-biological data repositories
Source: Life Sci Soc Policy. 2016 Jan 20;12:1. doi: 10.1186/s40504-016-0034-6 (PMC4720627; doi:10.1186/s40504-016-0034-6)
Supplement: Additional file 1: — Question guide. (PDF 323 kb) [file 40504_2016_34_MOESM1_ESM.pdf]

VIEWS AND PREFERENCES OF ALBERTA BIRTH COHORT PARENT PARTICIPANTS ON SECONDARY USE OF RESEARCH DATA

Question Guide

PRE-FOCUS GROUP (30 minutes prior to start of focus group)

- When participants arrive, the co-facilitator will go through the following with them:
  - *Signed copy of the informed consent form*
  - *Signed confidentiality agreement*
  - *Ensure understanding of conversation guidelines and focus group outline*
  - *Introduce Backgrounder document, including study contact information*
  - *Ensure knowledge of snacks/refreshments/washroom facilities' location*
  - *Answer any questions or concerns*

- Moderator will facilitate mingling and informal introductions among participants

WELCOME

- Welcome everyone. We want to thank you for agreeing to spend this time with us.

- Before we delve into our questions and main discussion, let's start with some brief introductions.
  - *We can stop at any time. We will be taping this focus group to make sure we note everyone's opinion accurately.*
  - *Does anyone have any questions before we start?*

OPENING QUESTION

- We'd like to start off with introductions.

- I would like us to go around the room. I would like you to say your name, tell us which study you are a part of (AOB or APRON) and tell us what was the most interesting summer job you ever had?

BACKGROUND

- Great! Let's start with our first topic, the re-use of research data.

- To start off, does anyone like to share what they think of when they hear "research"?
  - *What do you think research involves?*

- Why do you participate in research?

- First, everyone is here because you all share the common experience of being parent participants in a long-term cohort research study following both yourselves and your children.

- The information collected during a research study is often called data.

- *Give example of original and secondary question from same data set.*

- Researchers collect data to answer, or look at, specific research questions.
- But, once data is collected, it could be used (by itself or with data) to answer other, secondary questions.

- Research data repositories are akin to "data libraries". These libraries would enable sharing amongst qualified researchers. They act as a centralized system for collecting, storing and sharing research data from different studies. The goal is that this data becomes a legacy: a resource available for organized sharing for years or decades into the future.

- *Go over common language backgrounder.*
- *Please ask any questions as you have them.*
- *We will be covering a few different topics as we go along, but are there any preliminary questions.*

<u>INTRODUCTORY QUESTION</u>

- What are your general thoughts about sharing research data with other, qualified secondary researchers?

- *Thoughts on research data sharing? (or how do you feel about information sharing)?*
- *Tell me more about that?*

- WHAT COMES TO MIND… when you think of research data sharing?

- *Why do you feel like that?*

- WHAT COMES TO MIND… when you think of research data libraries?

- *Anything else?*
- *Who else has some general thoughts about sharing research data?*
- *What comes to mind?*
- *What thoughts do you have, if any, for the participants? Researchers?*

- Thoughts on research data library?

- *Tell me more about this?*
- *Why do you feel like that?*
- *Anything else?*
- *Who else has some general thoughts about research data libraries?*
- *What comes to mind?*
- *What do you think it involves? (Buildings? Personnel? Internet?)*

<u>BACKGROUND</u>

- I'd like you all to turn to the Backgrounder handout you were given when you arrived here today.

- In the Backgrounder, we introduce you to the proposed Research Data Repository of the Child Data Centre of Alberta. This data library aims to store and share research data on child development, health and wellbeing in Alberta.

- The goal of centralized data libraries are to create data legacies, so that the data will last and be shared over years or decades into the future.
- Many research funders now require that researchers make their data available to share with other researchers for re-use, or secondary data use. This is part due to a recognition of the significant public investment in research, and data collection. The push for sharing data is clear. But, what is unclear is the process of data sharing.

- *There is international agreement that re-use has many benefits. These benefits include an increase in the value for money spent on research, the ability to best use the gift of data participants have given; and an increase in collaboration amongst researchers in different disciplines.*

- We want your important input, as parent participants, on some of the processes planned for the Research Data Laboratory

- Your input will help make sure the process of data sharing is done thoughtfully and appropriately.

- Let me also remind you that nothing you say today will bind you on any future questions we ask or decisions. In particular, we are not asking you to decide whether or not you will share your, or your child's, data with the Research Data Repository

KEY QUESTIONS

- The Backgrounder provides details on the different protective strategies planned for the Research Data Repository.

- Some strategies are internal, or structural, to the library.

- Other strategies are external, or more relational, to the library.

- These strategies were developed based on our understanding of current laws and guidelines, what is happening in other countries, and what the most state-of-the-art technology offers.

- We want your insight, especially as many of these strategies go beyond the protections of the traditional, single research project.

- What do you think of these protective strategies?

- What do you think of the ACCESS AGREEMENTS & PROCESSES strategy? (*Green boxes in Backgrounder*)

- *Thoughts on protective strategies?*
- *What do you see as the strengths of this strategy?*

- *What do you see as the weaknesses in this strategy?*
- *Do you think this strategy will be effective? Why or why not?*
- *What sanctions should breaches entail?*
- *What limits would you want on how data can be accessed? Used?*

- What do you think of the OVERSIGHT COMMITTEES & FRAMEWORK strategy? (*Maroon boxes in Backgrounder)*

- *What do you see as the strengths of this strategy?*
- *What do you see as the weaknesses in this strategy?*
- *Do you think this strategy will be effective? Why or why not?*
- *Who should be a part of these committees?*
- *What activities of the repository should be watched most closely? (e.g. getting data? Storing data? Sharing data? Results/impact of sharing data? Interacting with researchers? Interacting with participants? Interacting with for-profit researchers/companies?*

- What do you think of the PROVINCIAL CONNECTIONS strategy? (*Red boxes in Backgrounder)*

- *What do you see as the benefit of this strategy?*
- *What do you see as the weaknesses in this strategy?*
- *Do you think this strategy will be effective? Why or why not?*
- *What limits would you want on how data can be accessed? Used?*
- *Are there other organizations that should have formal links with the repository? Who?*

- What other types of protective strategies would you like to see?

- *Other strategies?*

- Is there anything missing from what has been described?

- Do you think there is any difference between child data and adult data? Why or why not?

- *Do you see any gaps in the process or in the ways that protection could be enhanced?*
- *What other aspects of the library's role should be controlled or overseen? Why?*
- *What other aspects of the data storage or management should be controlled? How?*
- *What other aspects of the secondary researchers should be monitored? How? Why?*

10 MINUTE BREAK - Let's break here for 10 minutes before we return to the discussion.

- Getting permission from research participants is a very important step before research data can be included in a data library and can be shared with other researchers.

- Generally called consent, permission gathering can take a few different forms.

- There are mainly five different ways to ask for permission to share data with a research data library, as shown in Table 2 of the Backgrounder.

- Using little case vignettes, we would like your opinions on the strengths and weaknesses on the different ways that families might asked for permission.

- In each vignette, we ask you to consider Sally and her 11 month-old daughter Lila. Both are participants in a long-term cohort research project called Alberta Babies Cohort (or ABC for short). ABC has followed them since Sally was 26 weeks pregnant with Lila. The research has collected questionnaire data on health, development, stress, environmental factors, nutritional intake, and emotional state. ABC has also collected blood samples.

- FIRST CASE: Sally is asked to share her and Lila's non-biological data from ABC with a provincial research data library on child health and development. If Sally shares with the data library, they will be contacted directly in the future each time a secondary researcher wants to use either Sally's or Lila's data.

- This is the TRADITIONAL, OPT-IN consent model:

- *What do you think of the traditional, opt-in consent model?*
- *What are the strengths of traditional, opt-in consent?*
- *What are the weaknesses of this model?*
- *What impact does this model have on costs (to participant, to researcher, to repository)? On time (participant, researcher, repository)?*
- *Do you feel this model respects participants? Why or why not?*
- *What, if anything, feels essential about this model?*
- *What, if anything, feels unnecessary or excessive?*
- *What do you think of Sally's concerns for herself compared to concerns for Lila?*

- SECOND CASE: Sally and Lila are asked to share their non-biological data from ABC with a research data library on child health and development. If Sally gives permission, the data library will then decide how, when and why their data will be shared with future, secondary researchers. The library will base its decisions on the protective strategies in place.

- This is the BROAD, OPT-IN consent model:

- *What do you think of the broad, opt-in consent model?*
- *What are the strengths of the broad, opt-in consent?*
- *What are the weaknesses of this model?*
- *What impact does this model have on costs (to participant, to researcher, to repository)? On time (participant, researcher, repository)?*
- *Do you feel this model respects participants? Why or why not?*
- *What, if anything, feels essential about this model?*
- *What, if anything, feels unnecessary or excessive?*
- *How does this model compare to the other one we discussed?*

- THIRD CASE: Sally and Lila are asked to share their non-biological data from ABC with a research data library on child health and development. If Sally gives permission, the data library will then decide how,

when and why their data will be shared with future, secondary researchers. The library will base its decisions on the protective strategies in place. Every two years, the library will re-contact Sally to ask for permission to continue storing and sharing her and Lila's non-biological data.

- This is the BROAD, PERIODIC consent model:
  - *What do you think of the broad periodic consent model?*
  - *What are the strengths of the broad periodic consent?*
  - *What are the weaknesses of this model?*
  - *What impact does this model have on costs (to participant, to researcher, to repository)? On time (participant, researcher, repository)?*
  - *Do you feel this model respects participants? Why or why not?*
  - *What, if anything, feels essential about this model?*
  - *What, if anything, feels unnecessary or excessive?*
  - *How does this model compare to the other ones we discussed?*

- FOURTH CASE: Sally and Lila are asked to share their non-biological data from ABC with a research data library on child health and development. If Sally gives permission, she will also give the data library a list of preferences on how her and Lila's data can be shared in the future. In particular, Sally will specify the type of research, the type of researcher, and the research context their data may be shared in. The data library will use this list of preferences and its protective strategies to decide how secondary researchers can access the data.

- This is the TIERED consent model:
  - *What do you think of the tiered consent model?*
  - *What are the strengths of the tiered consent?*
  - *What are the weaknesses of this model?*
  - *What impact does this model have on costs (to participant, to researcher, to repository)? On time (participant, researcher, repository)?*
  - *Do you feel this model respects participants? Why or why not?*
  - *What, if anything, feels essential about this model?*
  - *What, if anything, feels unnecessary or excessive?*
  - *How does this model compare to the other ones we discussed?*

- FIFTH CASE: Sally and Lila are sent a letter telling them that, in 3 months time, their non-biological data from ABC will be stored in a research data library focused on child health and development. The data library will share this data with qualified secondary researchers for new research. The library will decide access to the data using its protective strategies. If Sally does NOT want to give permission for this storage and sharing, she must contact the data library within the next 3 months. If Sally does nothing, she has given her permission.

- This is the OPT-OUT consent model:
  - *What do you think of the opt-out consent model?*
  - *What are the strengths of the opt-out consent?*
  - *What are the weaknesses of this model?*

- *What impact does this model have on costs (to participant, to researcher, to repository)? On time (participant, researcher, repository)?*
- *Do you feel this model respects participants? Why or why not?*
- *What, if anything, feels essential about this model?*
- *What, if anything, feels unnecessary or excessive?*
- *How does this model compare to the other ones we discussed?*

- In the context of thinking about these consent models, how do you view the concerns of Sally compared to the concerns of Lila? How did this impact your responses? How should/if/when Lila be involved in decision-making in the long-term?

- Hypothetically, if data collection and then sharing, continues to the teenage years, at what point should children's (e.g. Lila's) preferences be considered? How?

- What other issues or ideas come to mind when you think of permission granting by parents for themselves and their children for sharing research data?

- Of all these options, would anyone like to share which option they most prefer?
    - *Why? How did you come to that decision at this point in time?*

- Why? How did you come to that decision at this point in time?
    - *Why? How did you come to that decision at this point in time?*
    - *Would the option you prefer differ if it was only for your data? Only for your child's?*
    - *How (and when) do you think your children should be involved in the consent process for the re-use of their data?*
    - *Do you feel that one parent's permission for including the data about their underage child is enough? When two parents are "in the picture", should there be requirements to have both their permission?*

<u>ENDING QUESTIONS</u>

- All things considered, would you be willing to share your research data from the cohort with a research data repository? And remember, your response is in no way binding.
    - *Did the discussion today influence your willingness?*
    - *What was most influential in this decision?*
    - *After recap parent preference, looking at all models, what was most influential in your decision-making?*

- Moderator summarizes top 3-5 key points in the discussion. Emphasis on perceived risks, benefits, and any preferences amongst governance and protective strategies.
    - *OR MODERATOR notes some of the points of greatest discussion by the group as a recognition that we heard them.*
    - *Did I correctly describe what was said?*

- As you recall, the purpose of this study is to understand your perspective as parent participants about sharing your own and your child's non-biological research data in a research data repository. With that in mind, is there anything else you want to add to the discussion?

- *Have we missed anything?*
- *Is there anything else that we should have talked about but didn't?*
- *Is there a key point that was not emphasized as much as you would like?*
- *Would you be interested in future discussions of this type?*

- Here is a hand-out containing some information on the proposed research data repository that spurred this research. The sheet also contains contact information for our research team if you have any questions or concerns.

- Thank you for your time and thoughtful contributions!
