# Supplementary material for: Parental perspectives on consent for participation in large-scale, non-biological data repositories
Source: Life Sci Soc Policy. 2016 Jan 20;12:1. doi: 10.1186/s40504-016-0034-6 (PMC4720627; doi:10.1186/s40504-016-0034-6)
Supplement: Additional file 2: — Backgrounder document that detailed the types of internal and external regulation data repositories. (PDF 210 kb) [file 40504_2016_34_MOESM2_ESM.pdf]

BACKGROUNDER

The **Child Data Centre of Alberta** will be a data and research facility on child development, health and well-being. It will include a **library** for research data, called the **Research Data Repository** (RDR) and will:

- **Store and manage data** from different research studies.
- **Oversee** the **sharing** of the research data with qualified researchers, to study new research questions.
- The data will include group-level and individual-level data on information like demographics (e.g. age and gender), health, social factors, emotional factors, and development.
- All Our Babies and APrON studies contain lots of valuable data on over 5000 mothers and babies, and about 1100 fathers. **Research funders and researchers are encouraging the sharing of AOB and APrON** data in the RDR.

The process of data sharing will involve **<u>standards and procedures</u>**. These strategies will protect the interests of the original research participants and provide clear rules for who can access the data and how they can use it.

Protective Strategies Based INSIDE the Data Library

Protective Strategies Linked to OUTSIDE the Data Library

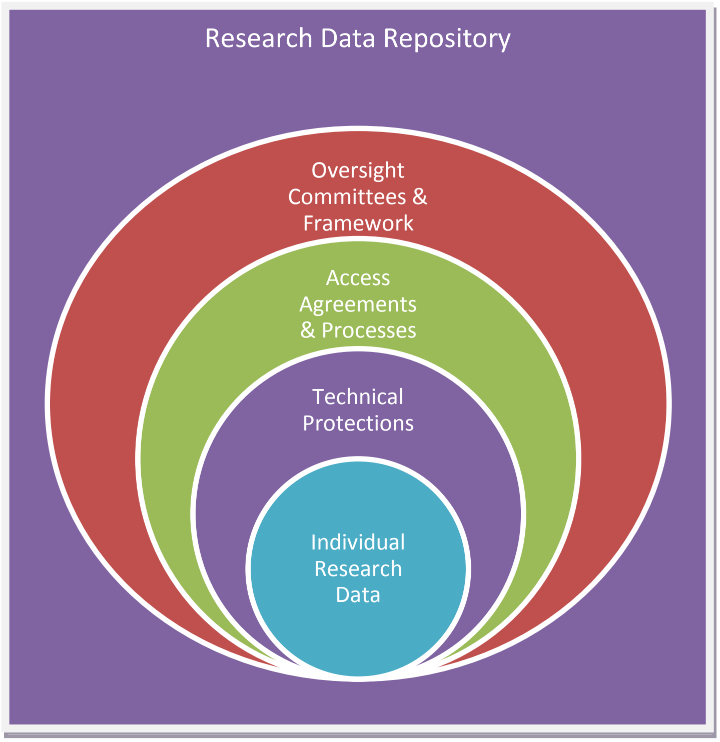

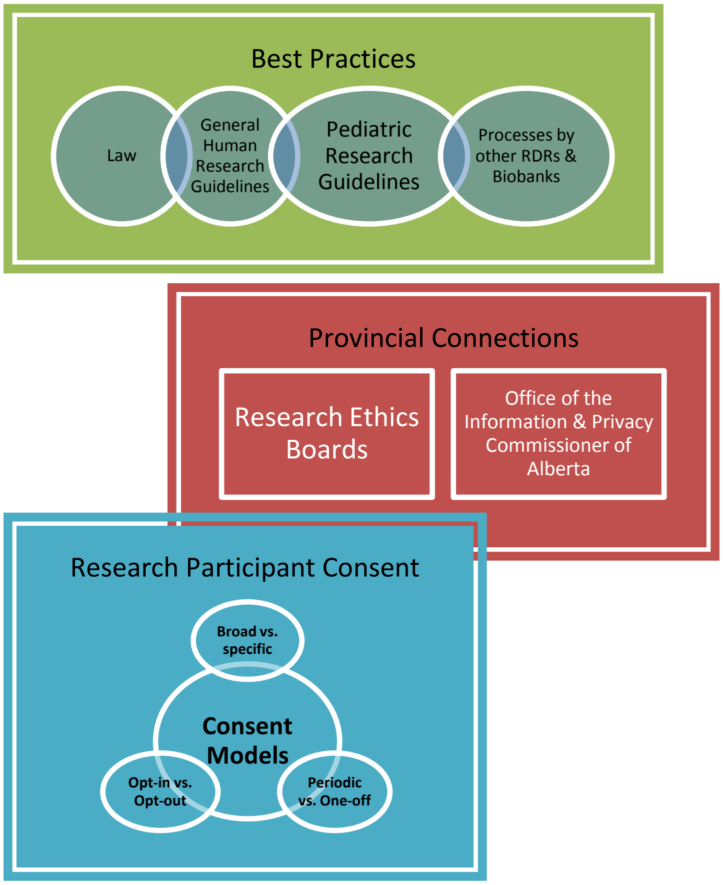

Details on Proposed Protective Strategies

Protective Strategy	Examples
**INSIDE DATA LIBRARY**	
**Technical Protections**	• Using most up-to-date technology to safeguard data before, during and after it is shared with researchers<br>• Providing data that is the minimum for the purpose, with identifying information removed to the maximum level possible
**Access Agreements & Processes**	• Researchers, and their proposed research projects, must meet qualification criteria<br>• Formal request process to get access to data<br>• Signed data sharing agreements that include requirements for using the data including penalties for misuse
**Oversight Committees & Framework**	• Committee including public and research participant members to oversee the set-up and operation of the data library<br>• Committee to review and approve data access requests
**OUTSIDE DATA LIBRARY**	
**Informed Consent**	• Getting permission (consent) from research participants to store, manage and share their research data<br>• Consulting with research participants on their preferred options for consent.
**Provincial Connections**	• Formal connection with Research Ethics Boards, to review both library structure and the conditions for transfer of data<br>• Formal connection with the Office of the Information & Privacy Commissioner of Alberta, for support and assessment around privacy protections
**Best Practices**	• Following the relevant laws, including the *Health Information Act*, and the *Freedom of Information & Protection of Privacy Act* and any future laws that impact data libraries.<br>• Following national standards on research ethics in Canada (the Tri-Council Policy 2), for human subjects and for pediatric research<br>• Modeling successful practices of other data libraries

Options for Participant Consent for Research Data Library

Note that these options are presented just for discussion and to get your opinions.
No decisions have been made about any particular consent approach for the Research Data Library as yet. However, your opinions (in addition to current ethical standards in Canada) will be very important in deciding what approach to use.

Consider Sally and her 11 month-old daughter Lila. Both are participants in a long-term cohort research project called Alberta Babies Cohort (or ABC for short). ABC has followed them since Sally was 26 weeks pregnant with Lila. The research has collected questionnaire data yearly on health, development, stress, environmental factors, nutritional intake, and emotional state.

The data library will have Sally's identifying information (e.g. name, address) but that information will not be available to new researchers. ABC has also collected blood samples but those will not be in the data library.

In each case below Sally has been asked to share her and Lila's data with a provincial research data library on child health and development.

Type of Consent	Definition	Case Vignette
A. Traditional, Opt-In Consent	Participants are asked for permission for all new uses of their data, including sending it to a library and every time a new researcher wants to access it.	Sally agrees to share data, and is contacted directly for consent every time a secondary researcher wants to use either Sally's or Lila's data in the future.
B. Broad, One-Time Consent	Participants are asked for permission for their data to go to the research data library just once, for similar purposes to the original use. After that one-time permission, all uses of the data are managed by the library's processes.	Sally agrees that her and Lila's data can go to the data library, and then the data library process determines who can access the data and how it must be protected each time.
C. Broad, Periodic Consent	Participants are asked for permission for their data to go to the library for future uses, but the library checks back with them every so often to see if they are still okay with their data being there and being shared.	Sally agrees that her and Lila's data can go to the data library, and then the data library process determines who can access the data and how it must be protected each time but also every two years, the library re-contacts Sally to ask for permission to continue storing and sharing her and Lila's non-biological data.

D. Tiered Consent	Participants are asked for permission for their data to go to the library for future uses. Participants are also asked for their preferences about the categories of future uses they will allow. For example, they may say they only want studies about certain topics. Or, they may say only student researchers can access their data. The library uses the list of preferences to decide how to share that participants' data.	Sally agrees that her and Lila's data can go to the data library but she also gives the data library a list of preferences on the type of researcher and the type of research that their data may be shared with and for. These choices are used in the access process.
E. Opt-Out Consent	Participants are informed that their research data will be placed in the library, and they can do nothing if they are okay with that. If they do not want their data to be included, they can answer the library within a set time period.	Sally is sent a letter telling her that, in 3 months time, her and Lila's non-biological data will be stored in the research data library. The library process will determine access If Sally does NOT want to give permission for this storage and sharing, she must contact the data library within the next 3 months. If Sally does nothing, this will be considered permission.
